# Comparison between Cashew-Based and Petrochemical Hydroxyoximes: Insights from Molecular Simulations

**DOI:** 10.3390/molecules28093971

**Published:** 2023-05-08

**Authors:** Cuong V. Nguyen, Chi M. Phan, Son A. Hoang, Shin-ichi Yusa

**Affiliations:** 1 Department of Water and Environmental Regulation, Joondalup, WA 6027, Australia; 2 Discipline of Chemical Engineering, Curtin University, Perth, WA 6845, Australia; 3 Institute of Materials Science, Vietnam Academy of Science and Technology, 18 Hoang Quoc Viet, Cau Giay, Ha Noi 11355, Vietnam; 4 Graduate School of Engineering, University of Hyogo, 2167, Shosha, Himeji 671-2280, Japan

**Keywords:** solvent extraction, copper complex, cardanol, cashew nut

## Abstract

Solvent extraction has been ubiquitously used to recover valuable metals from wastes such as spent batteries and electrical boards. With increasing demands for energy transition, there is a critical need to improve the recycling rate of critical metals, including copper. Therefore, the sustainability of reagents is critical for the overall sustainability of the process. Yet, the recycling process relies on functional organic compounds based on the hydroxyoxime group. To date, hydroxyoxime extractants have been produced from petrol-based chemical feedstocks. Recently, natural-based cardanol has been used to produce an alternative hydroxyoxime. The natural-based oxime has been employed to recover valuable metals (Ga, Ni, Co) via a liquid/liquid extraction process. The natural compound has a distinctive structure with 15 carbons in the alkyl tail. In contrast, petrol-based hydroxyoximes have only 12 or fewer carbons. However, the molecular advantages of this natural-based compound over the current petrol-based ones remain unclear. In this study, molecular dynamics simulation was employed to investigate the effect of extractant hydrocarbon chains on the extraction of copper ions. Two hydroxyoxime extractants with 12 and 15 carbons in the alkyl chain were found to have similar interactions with Cu^2+^ ions. Yet, a slight molecular binding increase was observed when the carbon chain was increased. In addition, lengthening the carbon chain made the extracting stage easier and the stripping stage harder. The binding would result in a lower pH in the extraction step and a lower pH in the stripping step. The insights from this molecular study would help design the extraction circuit using natural-based hydroxyoxime extractants. A successful application of cashew-based cardanol will improve the environmental benefits of the recycling process. With cashew-producing regions in developing countries, the application also improves these regions’ social and economic sustainability.

## 1. Introduction

Solvent extraction is an effective method of recovering divalent metal species from an aqueous solution. It has recently become a highly effective method of producing and purifying metals such as Ni, Co, Cu, and Mn, which are essential for energy production and storage. The process remains a critical step in recovering valuable metals from electronic wastes such as lithium-ion batteries and other electrical wastes [1]. The urgent need for an energy transition to a low-carbon economy requires a significant recycling effort. For instance, recycling is critical in providing copper for the transition to fossil-free electric energy. It has been estimated that photovoltaic power requires up to 40 times more copper than fossil fuel combustion [2]. To achieve the 2 °C scenario, 89% of the global identified copper resources will be extracted by 2050 [3]. Without effective recycling, the copper reserves (~870 Mt in 2019) would not be sufficient after 2040.

On the other hand, it is estimated that 45% of copper is currently disposed of in landfills and waste incinerators [4]. The percentage of collected and recycled metals is particularly low in developing countries due to a lack of technology, financial resources, and regulations [3]. It is estimated that a recycling rate of 70–90% is needed to achieve the 2 °C climate scenario. Such an ambitious recycling rate will require significant improvements in recycling processes. Consequently, there is an urgent need to develop an economical and practical process with potential application for localised and small-scale collection and recycling facilities. In addition to the mechanical collection and separation processes, the chemistry aspect of recycling also needs to be improved [5].

An environmentally friendly replacement of industrial chemicals, which are effective but expensive, is needed to improve sustainability. In addition to cheaper costs, alternative chemicals should have lower toxicity and lower risks in handling and transportation. Previously, organic and less hazardous acids, such as citric acid [6], malic acid [7], and oxalic acid [1], have been proposed to leach valuable metals from spent lithium-ion batteries. On the other hand, the production of extractants, such as hydroxyoximes, is exclusively based on alkyl phenols from petrol chemicals [8]. Finding a natural-based extractant can provide a significant pathway for copper recycling. The application of natural extractants is critical for the overall sustainability of the recycling processes.

In the copper and other transitional metal liquid/liquid solvent extraction processes, organic extractants play a critical role in separating valuable metals from other metals. The extractants, which have transferrable forming/breaking complexes with metal ions [9], are needed to transfer the target metal from the aqueous phase to the organic phase during the extraction step and from the organic phase to the aqueous phase in the stripping step. Among these organic compounds, the hydroxyoxime group (such as the Acorga and LIX series) is critical due to its capacity to form a simple complex with pH sensitivity. Copper and nickel extraction, for instance, rely mainly on these groups of extractants. Although some replacement attempts have taken place over a few decades, hydroxyoxime remains the only functional group for copper extraction [10]. These hydroxyoximes are currently synthesised via several reaction steps, such as oligomerisation and oximation [8]. The raw feedstock of the organic synthesis is alkyl phenol compounds, which are obtained from petrochemical processes. Since industrial alkyl phenol has 12 or fewer carbons in the alkyl branch [11], all current hydroxyoxime extractants have less than 12 carbons [12].

Recently, it has been found that natural cardanol, which can be readily obtained from cashew nutshell, can be used as an economical and environmentally friendly alternative to industrial alkyl phenol. The natural-based hydroxyoxime has been successfully used to extract gallium from bauxite liquor using kerosene as an organic phase [13]. The organic phase with 12% hydroxyoxime could extract 80% of gallium from concentrated Bayer liquor of an alumina processing plant. The cashew-based hydroxyoxime has also been used to extract nickel and cobalt from the mixture with manganese [14]. Most importantly, using natural-based compounds can reduce the carbon footprint of the industrial process from extractant production and the end-of-life phase. A sustainable replacement of petrochemicals is also a crucial strategy for reducing CO_2_ emissions [15].

**Figure 1:**
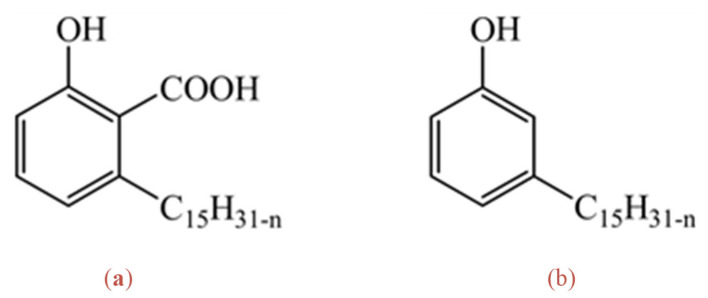
Two main components of the cashew-nut shell liquor, (**a**) anacardic acid, (**b**) cardanol.

It should be noted that the cashew tree (*Anacardium Occidentale*) is a valuable cash crop in tropical regions of Africa and Asia, where the economy is in the developing stage. Global cashew kernel production is around 700,000 metric tons per year [16]. Cashew shells make up a significant fraction (55–65% wt.) of cashew nuts. The natural liquor from cashew nut shells contains up to wt. 25% cardanol [17]. However, upon distillation at high temperatures (~190 °C), the cardanol content can be as high as 65% due to the decarboxylation of anacardic acid [18], as shown in Figure 1. In cashew nut processing, the shells are softened (to obtain the unbroken nuts) by steam. Consequently, the raw cashew nut shell liquor (CNSL) contains a high water content (~50% by weight). The raw CNSL is processed mechanically (pressing) and thermally (distillation to remove the water). The distillation process enhances the conversion of the anacardic acid to cardanol which can be used to synthesise oxime-based extractants. The current price of water-removed shell liquor is around USD 300–400 per metric tonne. With the expected climate changes in tropical regions, cashew cultivation suitability has been predicted to increase [19]. As far as we are aware, cashew nut shell liquor is the only natural source of alkyl phenols at these levels of abundance, chemical purity and affordability. Using this vast resource as a specialised chemical will enhance social-economic benefits for developing countries in these regions. It can enhance mineral recovery to realise environmental, social, and governance principles.

While the environmental benefits of cashew-based oxime are obvious, the molecular advantages of this compound over the current petrol-based oximes remain unclear. Previous equilibrium data have shown that the natural-based oxime has similar extraction efficiency to a commercial petrol-based extractant (LIX 860) [14]. Yet, the industrial application will also reply to the kinetics of elementary processes. The overall kinetics of solvent extraction depends on interfacial reactions and bulk transportation [20]. Danesi and co-workers [21] have qualitatively shown that the interfacial reaction can be the rate-determined step, especially at low aqueous ion concentrations. One of the distinctive characteristics of natural cardanol is the number of carbons in alkyl length. A previous study has shown that cashew-based cardanol has 15 carbons [18]. For amphiphilic molecules, it is well known that the alkyl length directly correlates to hydrophobicity and thus the molecular hydrophilic-lipophilic balance (HLB) [22]. For salicylaldehyde oximes, the increased alkyl length can decrease HLB by ~0.17 per ethylene group [23]. The molecular structure might provide additional impacts on the ionic transfer within the water/organic interface. However, the influence at a molecular level cannot be investigated via experimental methods. A clarification of the molecular role can enhance the industrial application of cashew-based reagents.

Consequently, this study used molecular simulations to provide new insights at the molecular scale. Ultimately, we aim to identify any potential benefits or/and shortcomings of the extra carbon length of the natural-based oxime in metal extraction.

## 2. Results

**Figure 2:**
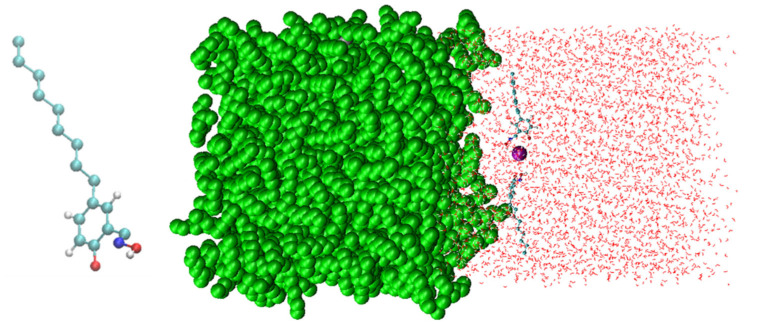
Simulation: (**left**) molecular structure of an extractant, (**right**) simulation box (red: water; green: hexane; purple: Cu^2+^).

In this study, the molecular interactions between hydroxyoximes and Cu^2+^ were investigated near the hexane/water interface. The oil/water interface was simulated by constructing a 5 × 5 × 5 nm box of water placed next to a 5 × 5 × 5 nm box of hexane, as shown in Figure 2 (right). Two extractant molecules (Figure 2 (left)) and a cation Cu^2+^ was placed in the aqueous phase close to the oil/water interface.

After the simulation (detailed in the Materials and Methods section), the molecular profile of the last 10 ns was used for density and radial distribution function (RDF) analysis.

### 2.1. Interfacial Molecular Structure

Figure 2 shows the density profiles of extractants and Cu^2+^ ions at the hexane/water interface. It is clear that the extractant’s tails reside in the oil phase while the polar heads are immersed in the water phase. The position of the O^−^ atom is unchanged relative to the interface between C_12_O_x_ and C_15_O_x_. However, the nitrogen of C12 is considerably closer to the interface than that of C15, revealing that changing the hydrocarbon chain length would alter the orientation of extractant molecules at the oil/water interface and therefore would change the transportation of metal ions in the extraction process.

It can be seen that Cu^2+^ ions reside at a similar distance from the oil/water interface despite altering the carbon chain length of the extractants. The distribution means that tail length has an insignificant effect on cation position. The carbon length also indicates a reasonable range for complex transfer between the two phases. Longer tails would reduce the solubility of extractants in the aqueous phase, whereas shorter tails would expel the complexation further into the water phase.

### 2.2. Coordination Structure of Extractant and Cu^2+^

A coordination structure of extractant complexes with divalent metal ions is dominated by four ionic bonds with O and N atoms of oxime groups [9]. The phenolic oxime compounds can coordinate well with Cu^2+^ cations (*r* = ~0.6Å [24]) in the aqueous solution for extraction activities. The selective formation and stability of the complexes are defined by hydrogen bonding between two bidentate units [25].

**Figure 3:**
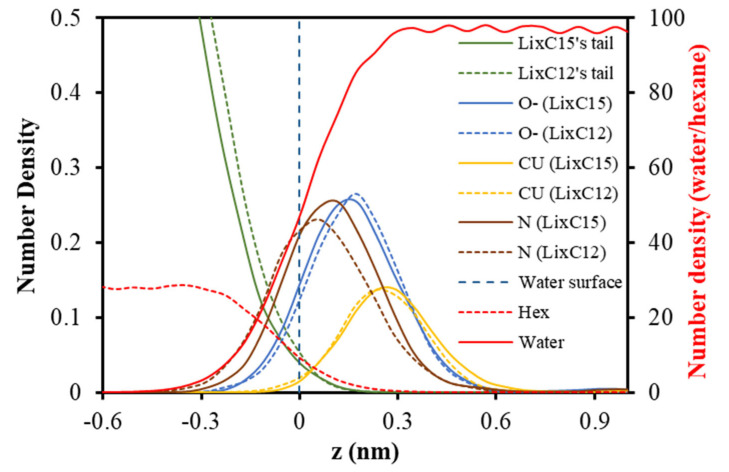
Density profiles of species at the hexane/water interface.

Consequently, the radial distribution function of O_p_ (that is, the oxygen atom on the phenolic ring), N, O, and H in the O-H group around the Cu^2+^ cation is plotted in Figure 3. For the longer extractant, the O^−^/Cu^2+^ binding is insignificantly different from the N/Cu^2+^ binding. On the other hand, the O^−^ of the extractant LixC12 can bind much stronger with Cu^2+^ cations. The N/Cu^2+^ binding follows an opposite trend: C12 has a considerably longer N/Cu^2+^ bond than LixC15. As the H atom (in the O-H group) of LixC12 resides further from Cu^2+^ cations with a substantially lower finding probability than that of C15, the hydrogen bonding in the LixC15/Cu^2+^ complex would be stronger than in the LixC12/Cu^2+^ one.

**Figure 4:**
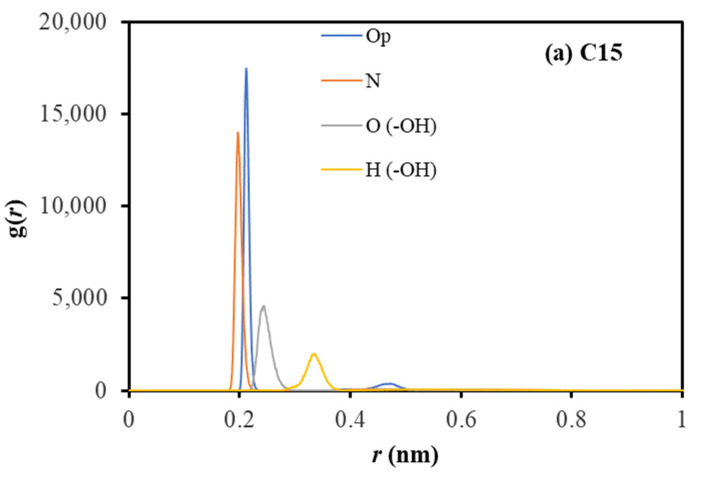

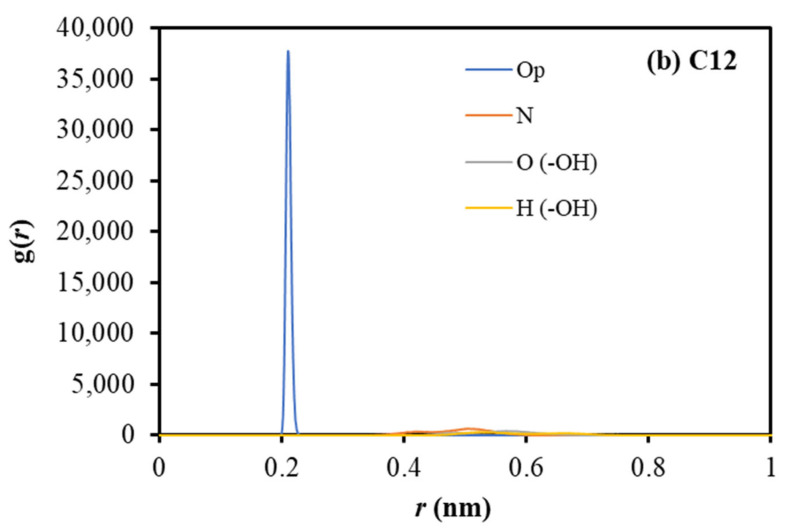
RDF of the head’s components around Cu^2+^.

In summary, the coordination revealed a clear impact of carbon length on the relative atomic position. In both cases, the O_p_/Cu^2+^ bonds are equally strong and should be the dominant bonds of metal-extraction complexes. For the longer chain, the N/Cu^2+^ has the same distance as O_p_/Cu^2+^ and thus provides four similar bonds (Figure 4a). For the short chain, the N/Cu^2+^ and other atoms are loosely bonded to the metal cation.

## 3. Discussion

The new insights at the molecular level can be used to predict the applicability of the new natural-based oxime in industrial processes. The overall kinetics of the solvent extraction process is controlled by chemical reactions and diffusion through interfacial film [26]. Consequently, the results are interpreted in these reaction kinetics and diffusion.

**Figure 5:**
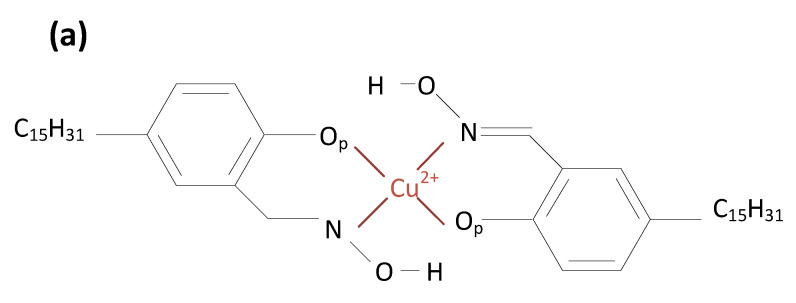

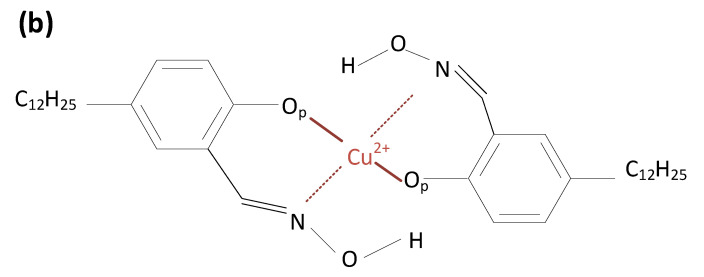
Extractant-Cu^2+^ coordination structure for (**a**) C15 and (**b**) C12 oximes.

**Figure 6:**
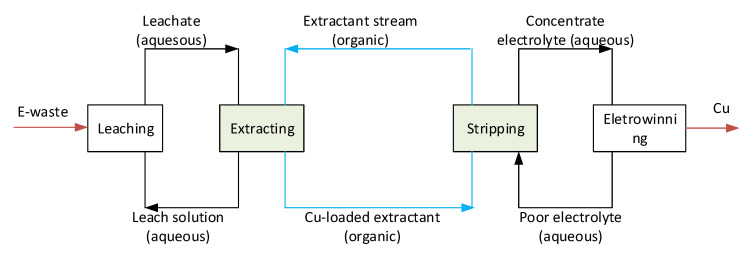
Recycling process to extract copper from e-waste.

The chemical reactions in solvent extraction include forming and dissociating metal complexes (Figure 5). It should be noted that the complete extraction circuit (Figure 6) includes both directions, i.e., extracting and stripping steps. While in the extracting (loading) step, metal complexes are transported from the aqueous phase (leachate) to the organic phase, the opposite transportation of the complexes from the organic phase to the aqueous phase occurs in the stripping step. The simulations showed that carbon length has a noticeable impact on ionic binding [27]. However, it does not alter the position of metal relative to the oil/water interface. The extra carbons in alkyl length reduce the flexibility of the extractant molecules. Similar effects have been reported in the literature. For instance, longer chain surfactants have a more rigid order at the oil/water interface [28]. A high density of the alkyl group also leads to a higher degree of molecular ordering and aggregation [29]. The molecular binding indicates that the C15 oxime might make the extracting stage easier and the stripping stage harder.

On the film diffusivity, it has been well reported that the self-diffusion coefficient of phenolic compounds is fairly constant [29]. Consequently, any advantages/disadvantages of the cashew-based hydroxyoxime should arise from the difference in the carbon chain length. The increased carbon chain reduces the self-diffusion coefficient. For example, the diffusion coefficient of alkyltrimethylammonium bromide (-TAB) was measured by analysing NMR signals from proton NMR self-diffusion experiments and it was reported that the coefficients of C_14_TAB, C_15_TAB, and C_16_TAB are 4.43 × 10^−10^, 4.07 × 10^−10^, and 3.79 × 10^−10^ m^2^/s, respectively [30,31]. The decrease in diffusion coefficient when increasing the carbon chain means that LIXC15 molecules are less flexible than LIXC12 molecules. As a result, the former molecules have stronger metal binding than the latter molecules.

It should be noted that the molecular simulations in this study are limited to the ionised state of the extractants, which is relevant to the extracting (or loading) stage. In contrast, the extractant is de-ionised during the stripping stage [9]. For adsorption at an air/water surface, it has been shown that the “hydrophilicity” difference between alkyl-phenol and alkyl-phenolate can be equivalent to four carbon atoms in the hydrophobic tail [22]. One may expect a similar balance between ionised and de-ionised oxime at an oil/water interface. However, such data would require delicate neutron reflectometry [32,33]. Szymanowski and co-authors have demonstrated a clear correlation between the HLB of oximes and copper extraction rate [23]. The adsorption data with simulations of extractants at different ionic states can describe the kinetics of the complete extracting/stripping cycles.

In addition to the reaction kinetics and diffusibility, the new insights are also valuable for other applications. For instance, the simulations can be extended to model novel processes, such as microchannel [34] and hollow-fibre membrane [35]. In comparison with conventional extraction circuits, micro-reactors have enhanced mass transfer and lower energy consumption. In this type of reactor, the mass transfer across the organic–aqueous interface is driven by molecular forces instead of mechanical forces in the conventional mixers/settlers. Consequently, the molecular structure of oxime can play an essential role in deciding the residential time.

Finally, the current process often requires a modifier, such as an alkylphenol, alcohol or ester [5]. The chemistry of the modifiers remains unclear, especially on the interaction between modifiers and extractants or extractant–metal complexes. For example, alcohols are stronger modifiers due to their association with metal complexes rather than their association with extractants [36]. However, alcohols are strong adsorbants at the interface (due to a strong hydrophobicity) and compete with extractants for adsorption area [37]. One of the interesting modifiers is the esters group, which is not a strong adsorbant at the oil/water interface. Yet, it has been argued that esters can inhibit hydroxyoxime adsorption at the water/oil interface [5]. Such molecular interaction can only be explored by molecular simulations.

In summary, the changes in molecular structure between cashew-based hydroxyoxime lead to a molecular shift in the oxime–metal complexes, especially on oxygen–metal bondings. The changes can be signified in the presence of modifiers. Molecular simulation in this study can be extended to the modifier-extractant systems. Significantly, the simulation can reveal the H-bond structure between modifier, water molecules near the interface, and metal-extractant complexes. In addition to Cu (II), the hydroxyoximes are also needed to recover Ga (III), Ni (II), Co (II), Pd(II), Pt (IV), Mo (VI), U (VI), Eu (III), and Th (IV) [9].

## 4. Materials and Methods

**Figure 7:**
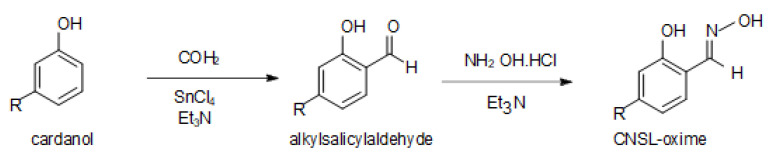
Reactions pathway to convert CNSL-cardanol to hydroxyoxime.

Cardanol was received from a cashew nut shell processing facility in Binh Phuoc Province, Vietnam. The conversion of cardanol to hydroxyoxime was carried out through two reactions process (Figure 7).

For the first reaction, the procedure included several mixing steps. First, cardanol was mixed with trimethylamine, at a volume ratio of 3:2. The cardanol–trimethylamine mixture was stirred for 30 min and then added to an SnCl_4_/toluene mixture (SnCl4: toluene volume ratio of 1:4) at the volume ratio of 1:1. The combined solution was stirred for 30 min before adding a mixture of formaldehyde and methanol (formaldehyde:methanol volume ratio of 3:2). This solution was stirred constantly for 24 h at 25 °C. The resulting alkyl salicylaldehyde product was rinsed and filtered using toluene and then de-ionised water.

The second reaction is essentially an oximisation reaction of the obtained alkyl salicylaldehyde. Alkyl salicylaldehyde was dissolved into de-ionised water (1:1 weight ratio), mixed with the same amount of hydroxylamine hydrochloride, and stirred for 30 min. A mixture constituting triethylamine and methanol (trimethylamine to methanol volume ratio of 1:2) was added to the solution for the oximisation process [13]. The reaction was maintained for 6 hrs at 25 °C under constant stirring (at 200 rpm). The final hydroxyoxime product was filtered and heated at 80 °C for 30 h to remove the organic solvent.

The intermediate (alkyl salicylaldehyde) and final (hydroxyoxime) products were diluted in toluene at wt. 5% for infrared characterisation. The characteristic bands of the oxime group were confirmed at 1607 cm^−1^ (C=N-OH bond) and 3396 cm^−1^ (O-H bond) [13].

The molecular simulations were conducted using GROMACS version 4.5.5 [38]. The aim of the simulation is to generate the molecular trajectories using a time step of 1 fs. A well-accepted water potential SPC/E (extended simple point charge model) [39] was used to simulate water molecules. In the literature, the SPC/E water model was demonstrated to generate the best bulk water dynamics and structure [40]. The molecular potential for the extractant and ions (Cu^2+^) has been described with GROMOS96 [41] force field. All hydrogen atoms of the extractant, except H in the hydroxyl group, were united with the corresponding carbon. The length of the carbon chain was varied to investigate the effect of the molecular structure of phenol-oxime extractants on ion coordination formation. Two molecules with 12 (C_12_OX) and 15 (C_15_OX) carbons in the alkyl branch were generated.

A 5 × 5 × 5 nm box of 3598 water molecules was placed next to a 5 × 5 × 5 nm box of 509 hexane molecules to form a hexane/water interface. In addition, a Cu^2+^ ion and two extractant molecules were placed in the aqueous phase near the hexane/water interface. This setup allowed for investigating the adsorption of extractant molecules to the oil/water interface as well as the interaction between the extractant molecules and Cu^2+^ cation. The charge distribution of utilised molecules and ions is tabulated in Table 1. It should be noted that the oxygen atom of the phenol group has a partially negative charge of −0.5159. On the other hand, the oxygen atom of the oxime group (which is labeled O_p_) has a full negative charge.

The simulation procedure included the following main steps. Firstly, the system was simulated at constant temperature and pressure (298 K and 1 atm, respectively) using a Berendsen barostat with a relaxation time of 2 ps and a cut-off of 1.3 nm to reach an equilibrium stage. After the equilibrium stage, the *x*-, *y*-, and *z*-dimensions of the simulation box were adjusted accordingly. The simulation was then performed for 20 ns at constant temperature (298.15 K) and volume using a Nose–Hoover thermostat [42]. This simulation time has been widely applied in the literature to ensure the achievement of an equilibrium stage of small extractant and surfactant molecules. The last 10 ns of this production run were used for density and radial distribution function (RDF) analyses. The geometry of the water molecules and bond lengths of extractant molecules were kept unchanged by employing LINCS [43] algorithms. Ewald [44] sums were used to deal with the electrostatic interactions. The density and RDF were obtained by analysing the trajectories recorded every 500 fs. Density and RDF were calculated via functions supplied with GROMACS.

Note that the obtained density profiles describe the number density of groups across the simulation box, which is useful for looking at the distribution of groups or atoms across the interface. The radial distribution function program divides the system into spherical slices around centre particles and computes a histogram. As a result, it describes how the density of surrounding atoms/groups varies as a function of the distance from a centre point.

## 5. Conclusions

The molecular simulation was carried out for extractant/copper interaction near an oil/water interface. The molecular insights indicated that the two molecules, with 12 and 15 carbons in alkyl length, have similar interactions with Cu^2+^ ions. The natural hydroxyoxime (15 carbons) does not alter the position of metal from the oil/water interface. However, natural hydroxyoxime has a stronger extractant–metal molecular binding. The increment indicated that the natural-based hydroxyoxime could interact strongly with metal ions. However, the shift in terms of molecular binding might make the extracting stage easier and the stripping stage harder. Hence, one can expect the equilibrium pH of natural-based hydroxyoxime to be lower during extraction and lower during the stripping stage. The simulations can be expanded to model the molecular interaction between modifiers and extractant–metal complexes. The simulation results might be useful in designing the extraction circuit based on the natural-based hydroxyoxime.

In conventional solvent extraction, which relies on emulsification and dispersion, the kinetics of the interfacial reaction is not a critical factor in overall efficiency. However, new extraction processes have been explored to reduce environmental impacts. For instance, a hollow membrane has been proposed [45] to reduce water, solvent, and energy usage. This new process can be applied on a smaller scale than in a typical metallurgical plant. In this process, the interfacial reaction plays a more critical role [35]. One of the potential applications of this study is that cashew-based hydroxyoxime can be incorporated within membrane extraction. This design would enhance the extraction of cation ions and simplify the stripping process. Note that the interaction between extractants and solvent plays a critical role in the extracting and stripping processes [46]. Therefore, the simulation in this study can be extended to investigate the interaction between cashew-based oxime and other solvents.

## Figures and Tables

**Table 1:** Charge distribution for simulation molecules and ions.

United Atoms/Ions	Charge
CH_x_ (Hexane)	0.0000
CH_x_ (CnOX)	0.0000
CH (=N)	0.3381
N	−0.2845
O (-OH)	−0.5159
H (-OH)	0.4623
O_p_	−1.0000
Cu^2+^	2.0000

## Data Availability

The data presented in this study are available on request from the corresponding author.

## Sample Availability

Samples of the synthsised oximes are available from the authors.
